# An TRIM59‐CDK6 axis regulates growth and metastasis of lung cancer

**DOI:** 10.1111/jcmm.14052

**Published:** 2018-12-04

**Authors:** Biao Geng, Manman Liang, Lilong qin, Wenying Zhao, Hanli wang, Lijing wang, Xianhui pan, Xingwu Chen

**Affiliations:** ^1^ Department of Respiratory Medicine Yijishan Hospital Wannan Medical College Wuhu Anhui China; ^2^ Department of Infectious Diseases Yijishan Hospital Wannan Medical College Wuhu Anhui China; ^3^ Department of Medical Oncology Yijishan Hospital Wannan Medical College Wuhu Anhui China

**Keywords:** CDK6, EMT, ERK pathway, lung cancer, TRIM59

## Abstract

Lung cancer (LC) is a devastating malignancy with no effective treatments, due to its complex genomic profile. Using bioinformatics analysis and immunohistochemical of lung carcinoma tissues, we show that TRIM59 as a critical oncoprotein relating to LC proliferation and metastasis. In this study, high TRIM59 expression was significantly correlated with lymph node metastasis, distant metastasis, and tumour stage. Furthermore, up‐regulation of TRIM59 expression correlated with poorer outcomes in LC patients. Mechanistically, TRIM59 play a key role in promoting LC growth and metastasis through regulation of extracellular‐signal regulated protein kinase (ERK) signalling pathway and epithelial‐to‐mesenchymal transition (EMT)‐markers, as validated by loss‐of‐function studies. In‐depth bioinformatics analysis showed that there is preliminary evidence of co‐expression of TRIM59 and cyclin dependent kinase 6 (CDK6) in LC. Notably, CDK6 expression significantly decreased when TRIM59 was knocked down in the LC cells. In contrast, exogenous up‐regulation of TRIM59 expression also induced significant increases in the expression of CDK6. Moreover, the expression of CDK6 was also inhibited by the ERK signalling inhibitor, U0126. The results of both loss‐ and gain‐of‐function studies showed that TRIM59 could regulate the expression of CDK6. Collectively, these data provide evidence that TRIM59 is involved in lung carcinoma growth and progression possibly through the induction of CDK6 expression and EMT process by activation of ERK pathway.

## 1 INTRODUCTION

Lung cancer (LC), the leading cause of cancer‐related death worldwide, is characterized by a high metastatic capacity. Diagnosis often occurs in late‐stage disease when most patients have missed the optimal window for surgery due to metastasis.^1^ Although significant improvements have been made in the prediction of LC outcomes using clinical information, such as TNM staging, the heterogeneity of outcomes observed among patients with similar phenotypic properties indicates that genetic variations may play crucial roles in LC growth and metastasis.^2^ The complicated molecular and cellular mechanisms involved in LC metastasis remain poorly understood. Thus, the need to identify potential therapeutic targets to improve LC treatment is urgent.

The tripartite motif (TRIM) family proteins, contain three highly conserved domains, which consist of a common N‐terminal Really Interesting New Gene finger domain, one or two B‐box motifs and a coiled‐coil region.^3^, ^4^ TRIM proteins are thought to be important regulators of carcinogenesis, which are involved in several biological processes, such as cell growth, development, and cellular differentiation and alteration of these proteins can affect transcriptional regulation, cell proliferation, and apoptosis.^5^ Previous studies have suggested that TRIM59 were increased significantly in tumour tissues compared with noncancerous tissues from glioblastoma and gastric tumours, where increased levels were associated with advanced tumour stage and shorter patient survival times.^6^, ^7^ Up‐regulation of the TRIM59 gene promotes gastric carcinogenesis via promoting the p53 ubiquitination and degradation.^6^ In addition, TRIM59 acts as a key oncogene by promoting EGFR/STAT3 signalling activation and mediates the development of proliferation and migration.^7^ Increasing evidence showed that TRIM59 has been implicated in mediating tumour progression. However, the mechanisms regarding how it facilitates tumourigenesis have not been elucidated.

Notably, silencing of TRIM59 significantly inhibited the proliferation and migration of non‐small cell lung cancer cells cell lines by arresting cell cycle in G2 phase.^8^ Cell cycle is gaining great attention in cancer research for their potential to regulate essentially every hallmark of tumour development. Yet the molecular mechanisms regulating and coordinating cell cycle remain. Bioinformatics analysis shows that expression of TRIM59 positively correlates with cyclin‐dependent kinases 6 (CDK6) in LC cell and tissue. The CDK 6 control the G1 phase of the cell cycle, which has a central role in cell proliferation and in tumourigenesis.^9^, ^10^ It is widely accepted that CDK6 is a leading gene relating to tumour growth and metastasis.^11^ Therefore, further investigation of the above association of TRIM59 and CDK6 is of pivotal importance.

In this study, we investigated the correlation of TRIM59 expression and the clinicopathological parameters in lung carcinoma and determined whether TRIM59 was involved in the epithelial‐mesenchymal transition (EMT) process of LC by activation of the extracellular‐signal regulated protein kinase (ERK) pathway. Furthermore, we further investigated the mechanisms that TRIM59 positively regulates CDK6 expression by activation of ERK pathway, which contribute to cancer cell growth and invasion.

## 2 MATERIALS AND METHODS

### 2.1 Cell cultures

The human LC cell lines H441, SPC, H292, A549, H1299, and H460 were purchased from the Chinese Academy of Science Cell Bank (Shanghai, China). Cell lines were routinely checked for contamination by Mycoplasma, using Hoescht staining, and were authenticated by DNA‐Fingerprinting and isoenzyme analyses. Both cell lines were maintained in RPMI1640 (Gibco, USA) supplemented with 10% foetal bovine serum (Gibco) and 1% penicillin/streptomycin (Corning, Lowell, MA, USA) at 37°C in a humidified incubator with 5% CO_2_.

### 2.2 Western blot analysis and antibodies

Western blot analysis of whole‐cell protein lysates was performed using primary antibodies against TRIM59 (Catalog no. PA5‐38726; Invitrogen), E‐cadherin (Catalog no. 14472; Cell Signaling Technology [CST]), N‐cadherin (Catalog no. 13116; CST), Snail (Catalog no. 3879; CST), Slug (Catalog no. 9585; CST), Vimentin (Catalog no. 5741; CST), CDK6 (Catalog no. 3136; CST), Ki‐67 (Catalog no. 9449; CST), Phospho‐p44/42 mitogen‐activated protein kinase (MAPK) (Erk1/2) (Thr202/Tyr204) (Catalog no. 4370; CST) p44/42 MAPK (Erk1/2) (Catalog no. 4695; CST), and a secondary anti‐mouse or anti‐rabbit IgG antibody. Equal amounts of protein, which were blotted with an anti‐β‐actin antibody (Catalog no. 3700; CST), were used as loading controls.

### 2.3 Immunohistochemistry

The incubated slides were then deparaffinized in xylene and rehydrated with graded alcohol. Next, antigens were retrieved using citrate buffer (pH 6.0). The samples were covered with 10% normal goat serum in phosphate buffered saline (PBS) for approximately 10 minutes at room temperature and then incubated with anti‐TRIM59 (Catalog no. PA5‐38726; Invitrogen) at 4°C overnight. For immunohistochemical detection an HRP‐Polymer Detection Kit (Abcam) followed by a DAB Substrate Kit (Abcam) were used, and slides were subsequently counterstained with haematoxylin. The percentage of TRIM59‐positive cells was scored in five groups: 0 (0% to ≤25%), 1 (25% to ≤50%), 2 (50% to ≤75%), and 3 (>75%). The 0 and 1 groups were defined as low expression, while 2 and 3 groups were defined as high expression.

### 2.4 Transwell invasion assay

Matrigel Basement Membrane Matrix (BD Biosciences) was used to determine the effect of cells on invasion according to the manufacturer's instructions. A total of 5 × 10^4^ cells were resuspended in basic RPMI‐1640culture medium and added in 200 μL to the upper chamber of a transwell system (8 μm pore size; Corning), while the lower chambers were filled with 500 μL RPMI‐1640 medium containing 10% FBS that served as a chemoattractant. After incubation for 24 hours at 37°C, invasive cells were fixed with 100% methanol and stained with 1% crystal violet solution before counting under an inverted microscope. All the experiments were done in duplicate, and results were expressed as mean ± SEM of three independent experiments.

### 2.5 RNA extraction and real‐time PCR

Total RNA was isolated using TRIzol reagent (Invitrogen) according to the manufacturer's protocol. For first‐strand cDNA synthesis, 500 ng of total RNA was retrotranscribed using Prime Script RT reagent Kit (Thermo Fisher Scientific). The resultant cDNA was subsequently amplified with the Maxima SYBR‐Green/Rox qPCR Master Mix 2X kit (Thermo Fisher Scientific) using the StepOnePlus Real‐Time PCR System (Thermo Fisher Scientific). All reactions were run in triplicate. Changes in the expression of CDK6 gene was determined relative to the mean critical threshold (CT) values of GAPDH gene. The primer pairs used were as follows: CDK6, 5′‐CGGGATCCACCATGGAGAAGGACGGCCTG‐3′ (forward) and 5′‐CGGATCCATTGCTCAGGCTGTATTCAGCTCCGA‐3′ (reverse); and GAPDH, 5′‐CTCTGCTCCTCCTGTTCGAC‐3′ (forward) and 5′‐CGACCAAATCCGTTGACTCC‐3′ (reverse).

### 2.6 Cell proliferation

Cell proliferation was assessed using Cell Counting Kit‐8 (CCK8) (DOJINDO) assay according to the manufacturer's instructions. Absorbance values were measured at the wave length of 450 nm as representation of cell viability. DNA synthesis was analysed using a Cell‐Light EdU Apollo488 In Vitro Imaging Kit (RiboBio) per the manufacturer's instructions.

### 2.7 Reagents

U0126 (mitogen‐activated protein kinase inhibitor, #S1901) was purchased from Beyotime (China). Stable TRIM59 was up‐regulated using Full length Clone DNA of Human TRIM59 with N terminal GFP Spark tag (Cat: HG25849‐ANG; Sino Biological Inc.) as per the manufacturer's instructions.

### 2.8 Bioinformatics analysis

The expression levels of genes were investigated in paired LC tissue samples based on Oncomine datasets (https://www.oncomine.org/). Co‐expression data were from Oncomine datasets. We used the following filters: gene “TRIM59” Analysis Type: “Co‐expression analysis” Cancer Type: “LC.”

### 2.9 Construction of H1299 and A549 TRIM59‐knockdown cells

Human TRIM59 shRNAs (Sigma) were used to knock down TRIM59 according to the protocols provided by the manufacturer. Cells were seeded on six‐well plates and transfected the next day with TRIM59 or control shRNAs. The cells were collected at the indicated time points and were subjected to western blot evaluations. Western blot evaluations using anti‐TRIM59 antibody demonstrated that these shRNA had over 90% of silencing efficacies for TRIM59.

### 2.10 Wound closure assay

Cells were allowed to form a confluent monolayer in a 24‐well plate. The wound was created by scraping a conventional pipette tip across the monolayer. Cells were washed with PBS, cultured in serum‐ and antibiotic‐free media at 37°C, and photographed at 0 and 24 hours.

### 2.11 Tumour xenograft transplantation assay

Subcutaneous xenografts were created in the flank regions of 4‐week‐old female BALB/c nude mice. A total of 5 × 10^6^ A549 cells stably expressing scrambled shRNA or shTRIM59 were implanted. In addition, a total of 5 × 10^6^ H460 cells stably transfected with pCMV3‐GFP‐TRIM59 or pCMV3‐GFP‐N1 (Vector) were independently injected subcutaneously into nude mice. Monitoring of tumour nodules was performed and tumour volumes were estimated with the following formula: Volume = width × length × (width + length)/2. The mice were euthanized on day 30 or 28 and tumours were taken. The animal studies were approved by the Wannan Medical College Ethics Review Board.

### 2.12 Statistical analysis

All calculations were performed with the SPSS 20.0 software program (SPSS Inc, Chicago, IL, USA). The relationship of TRIM59 expression and clinicopathological parameters was evaluated with chi‐squared test. The Kaplan‐Meier method was used to analyse patient survival. Student's *t* tests were used to evaluate continuous variables between subgroups. Statistical significance was defined as *P* < 0.05.

## 3 RESULTS

### 3.1 Clinical significance of TRIM59 in LC

**Figure 1:**
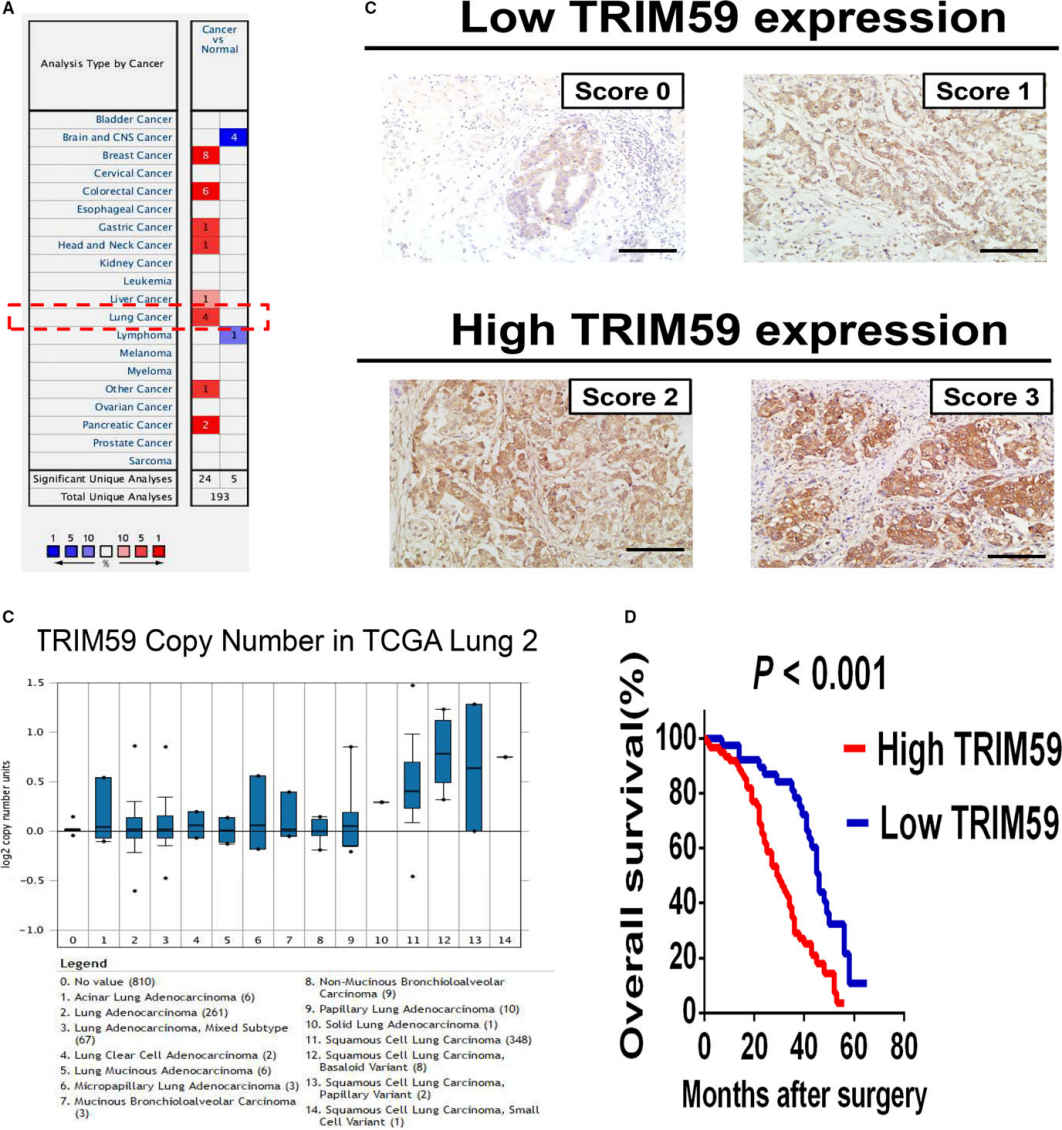
TRIM59 is overexpressed in lung cancer and is correlated with poor survival. (A) Up‐regulation of TRIM59 was found in 8 of 20 cancer types. (B) The TRIM59 expression is up‐regulated in lung cancer tissue compared with normal lung tissue as revealed by Oncomine data‐mining analysis in TCGA Lung 2's dataset. (C) Scores indicate TRIM59 levels in representative tumour tissues. The scores were calculated by intensity and percentage of stained cells as described in the materials and methods. Scale bars, 100 μm. (D) Kaplan‐Meier survival curves for overall survival (OS) in lung cancer patients according to TRIM59 expression. Patients with high TRIM59 expression (score 2‐3) have poorer overall survival compared with patients with low TRIM59 expression (score 0‐1)

To identify TRIM59 aberrantly expressed in LC, we first analysed all the differentially expressed TRIM59 within the microarray dataset from the Oncomine database. The results showed that TRIM59 expression was significantly higher in LC tissues compared to adjacent noncancerous tissues (Figure 1A,B). To further determine the above correlations of TRIM59 and clinical characteristics as well as the prognostic value of TRIM59 in LC, tissue microarray‐based IHC study of TRIM59 in 92 LC tissues with comparable clinicopathological features and complete follow‐up data were performed. Univariate analysis of 92 cases of LC demonstrated that TRIM59 expression levels was significantly correlated with lymph node metastasis, distant metastasis, and TNM stage (Table 1). To clarify the prognostic value of the TRIM59 among LC patients, the relation between their expression and survival time was next analysed by Kaplan‐Meier survival analysis. The results indicated that high expression of TRIM59 was positively correlated with poor prognosis. (Figure 1D). Therefore, our data suggested that TRIM59 overexpression is significantly associated with poor prognosis in LC patients.

**Table 1:** Analysis of association between TRIM59 expression and clinicopathological parameters in lung cancer

Characteristics	N	TRIM59 expression		*P*‐value
		Low	High	
Age				
≤60	33	19	14	0.161
>60	59	25	34	
Gender				
Male	54	28	26	0.357
Female	38	16	22	
Differentiation				
Well	13	8	5	0.555
Moderate	54	25	29	
Poor	25	11	14	
Tumor size (T)				
T1‐T2	29	18	11	0.064
T3‐T4	63	26	37	
Lymph node metastasis				
N0‐N1	31	21	10	0.006
N2‐N3	61	23	38	
Distant metastasis (M)				
Negative (M0)	83	43	40	0.032
Positive (M1)	9	1	8	
Tumor stage				
I‐II	43	26	17	0.023
III‐IV	49	18	31	

*P*‐values <0.05 were considered statistically significant (chi‐squared test for categorical variables).

### 3.2 TRIM59 promotes LC growth and metastasis

**Figure 2:**
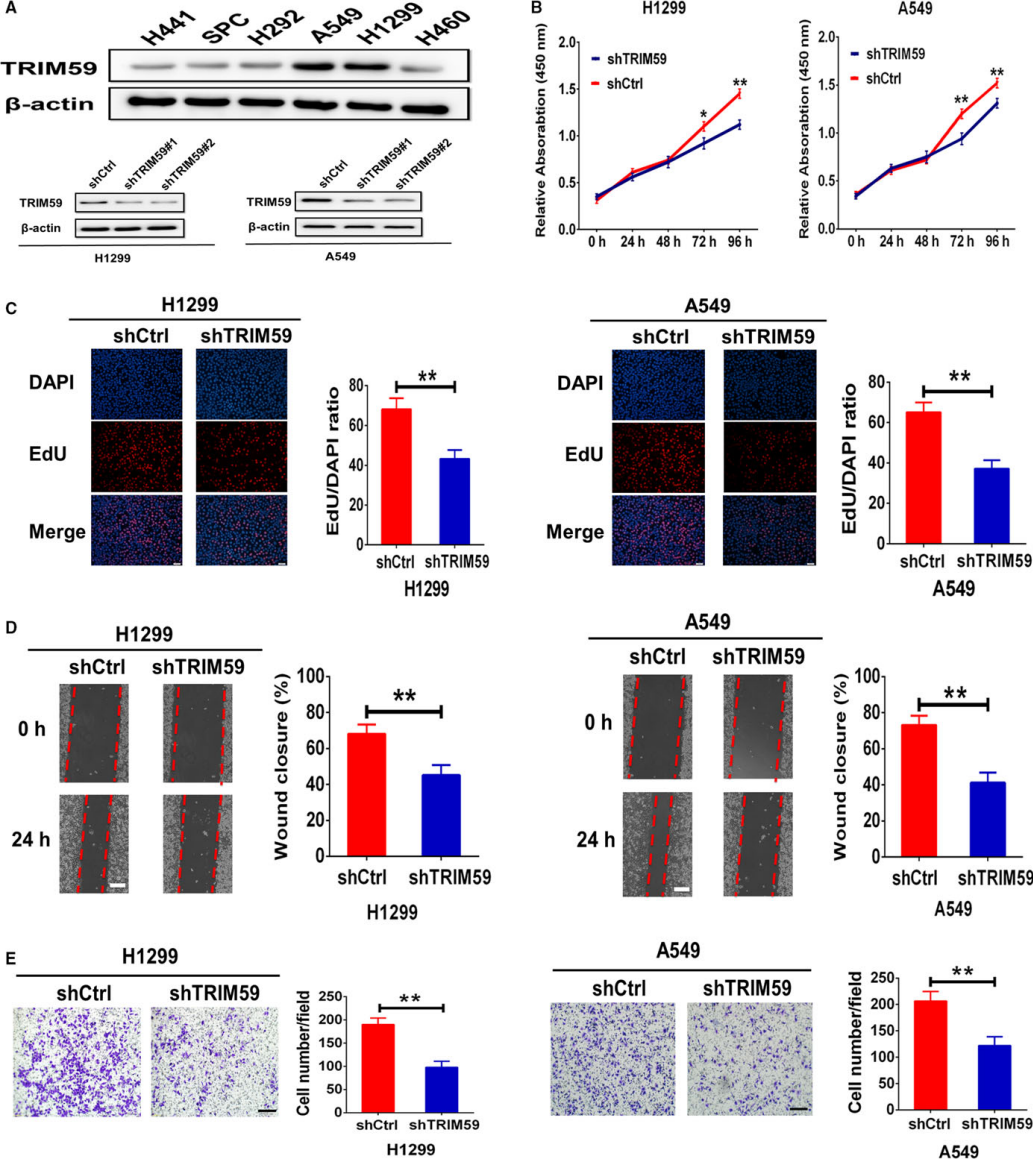
Roles of TRIM59 in promoting lung cancer growth and metastasis. (A) Expression levels of TRIM59 in the indicated lung cancer cell lines were analysed by western blotting (upper). Confirmation of TRIM59 knockdown in A549 and H1299 cells (lower). (B) CCK8 assays were used to analyse proliferation of cells stably transfected with Scrambled shRNA (shCtrl) or TRIM59 shRNA (shTRIM59). (C) EdU incorporation assay was used to analyse DNA synthesis rates of each transfected cell population. Scale bars, 100 μm. (D, E) Invasion of lung cells stably infected with shCtrl or shTRIM59 was assessed by the transwell invasion assays (scale bars, 100 μm) and wound‐healing assays (scale bars, 200 μm). Error bars indicate means ± SEM. All these experiments have been repeated three times. The significance was determined by Student *t* test. *P < 0.05; **P < 0.01

The above data showed that TRIM59 positively correlates with a more aggressive tumour phenotype. Accordingly, we investigated the functions of TRIM59 in LC cell lines using in vitro assays. In order to assess the effects of TRIM59 on in vitro cell growth and invasion, we first examined the endogenous TRIM59 levels of different LC cell lines and regulated their TRIM59 expression levels via stably transfecting TRIM59‐specific short hairpin (sh) RNAs in these cells. We found significantly increased TRIM59 levels in A549 and H1299 cell lines (TRIM59 high) (Figure 2A). To test the biological function of TRIM59 in LC progression, we carried out loss‐of‐function studies using H1299 and A549 cells as models to generate stable TRIM59‐Knockdown cell lines (Figure 2A). As shown in Figure 2B,C, Stable depletion of TRIM59 with shRNA in H1299 and A549 cells strongly inhibited proliferation rate.

**Figure 3:**
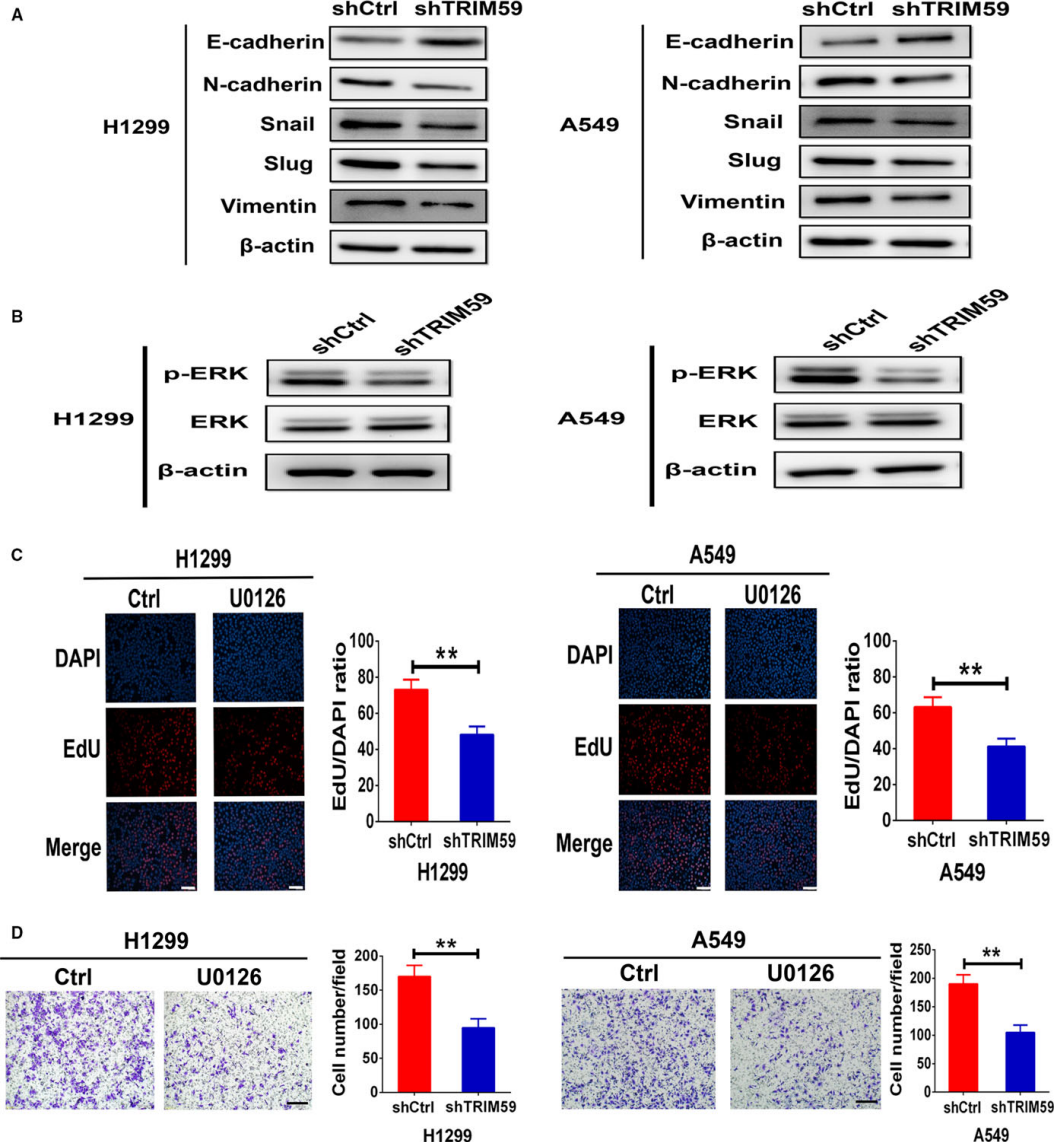
TRIM59 induced EMT program and ERK phosphorylation. (A) H1299 and A549 cells tranfected with shCtrl or shTRIM59 plasmid were subjected to western blotting for detection of E‐cadherin, N‐cadherin, Snail, Slug, and Vimentin expression. (B) Western blotting measurement of activation of ERK signaling by TRIM59 knockdown in H1299 and A549 cells. (C) DNA synthesis of H1299 and A549 cells treated with negative control and U0126 (10 μmol/L) was measured using an EdU‐incorporation assay. Scale bars, 100 μm. Results are expressed as means ± SEM. Data were assessed by Student's *t* test. *P < 0.05; **P < 0.01. (D) In vitro invasion assays using transwell chambers in H1299 and A549 cells treated with negative control and U0126 (10 μmol/L). Scale bars, 100 μm. Values are means ± SEM from three independent experiments. Significance was determined by Student's *t* test

In addition, scratch wound assays showed that wound recovery was significantly impaired by TRIM59 knockdown in comparison with the controls (Figure 2D). Moreover, Matrigel invasion assays verified the result, which showed that TRIM59 down‐regulation also induced strong inhibition on invasion of H1299 and A549 cells (Figure 2E). Thus, TRIM59 appears to be the critical oncoprotein in regulating tumour cell invasion. It is well‐known that the epithelial‐to‐mesenchymal transition (EMT)—a fundamental cell‐biological process that plays key roles in cancer cell invasion and metastasis.^12^ This prompted us to investigate the effects of TRIM59 down‐regulation on EMT‐markers expression. As expected, Western blot analysis showed that TRIM59 down‐regulation led to the up‐regulation of E‐cadherin and down‐regulation of N‐cadherin, Vimentin, Snail, and Slug in both H1299 and A549 cells (Figure 3A). These experiments provide strong evidence that TRIM59 potentially promotes EMT process in LC cells. Together, these data show that TRIM59 is the major factor that promotes LC cell growth and invasion.

### 3.3 TRIM59 induced ERK phosphorylation

Mitogen‐activated protein kinase signalling pathway has emerged as a central feature of EMT process.^13^ Furthermore, accumulating evidence indicates that ERK pathway play an critical role in LC cell growth and invasion through EMT.^14^, ^15^ The role of TRIM59 and involvement of EMT in growth and invasion led us to test this pathway. Thus, we investigated the effects of TRIM59 down‐regulation on p‐ERK and ERK expression, the key downstream effectors of the MAPK signalling pathway. Stable depletion of TRIM59 with shRNA in H1299 and A549 cells strongly inhibited ERK activation (Figure 3B). To test whether TRIM59 promote cancer cell growth and invasion through regulation of the ERK signalling pathway, we further examined the effects of ERK pathway activation on tumour proliferation and invasion of H1299 and A549 cells. These experiments showed that the proliferation and invasion of H1299 and A549 cells were strongly suppressed by ERK inhibitor U0126 at 10 μmol/L (Figure 3C,D). These data indicate that TRIM59 induced cancer cell growth and invasion possibly through regulation of the ERK signalling pathway.

### 3.4 TRIM59 induced up‐regulation of CDK6 expression is dependent on ERK pathway

**Figure 4:**
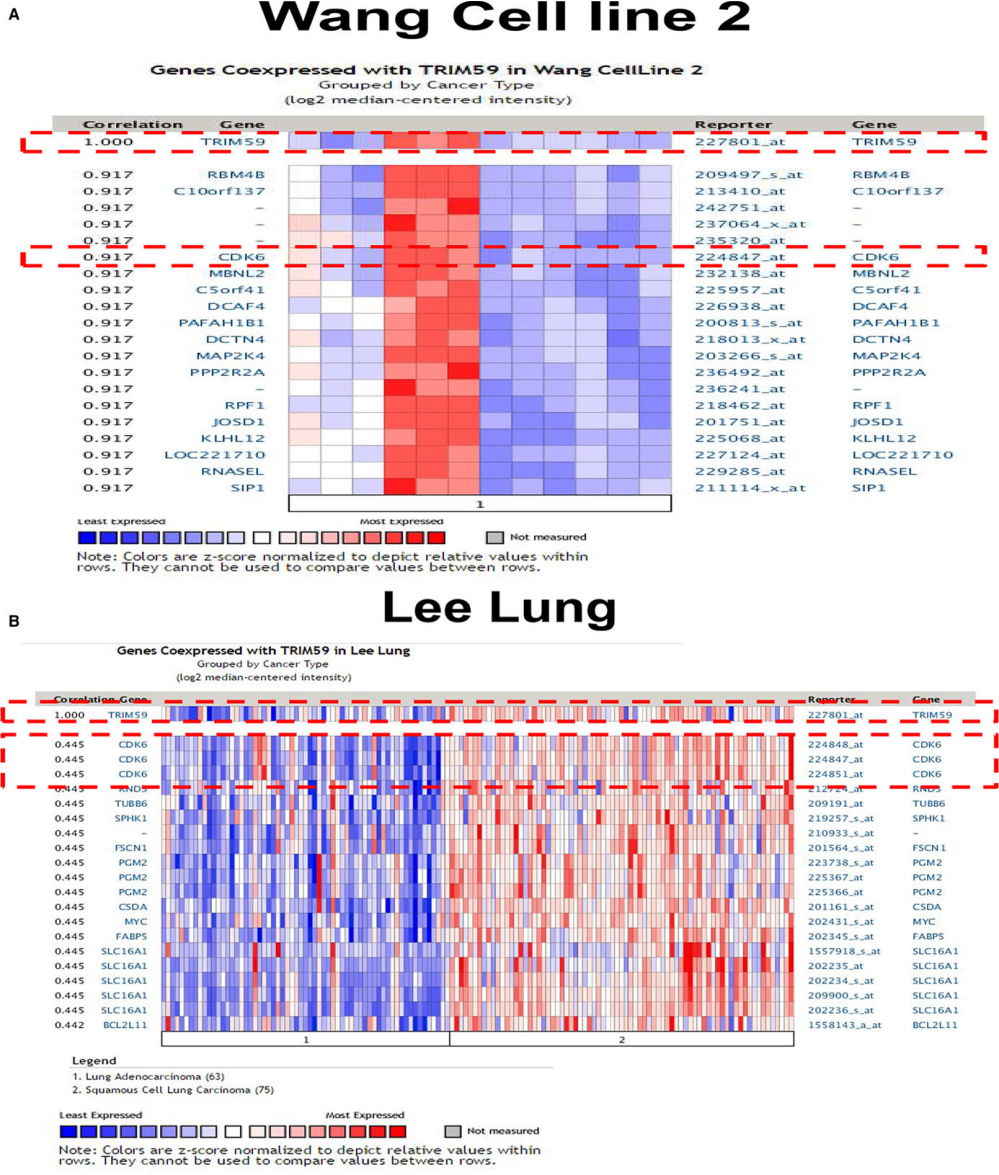
Coexpression of TRIM59 and CDK6 in lung cancer. (A, B) Co‐expression data from Oncomine (www.oncomine.org). We used the following filters: gene “TRIM59” Analysis Type: “Co‐expression analysis” Cancer Type: “Lung cancer.” The colour changed according to a weaker (blue) or higher (red) expression in wang cell line 2 and Lee lung

**Figure 5:**
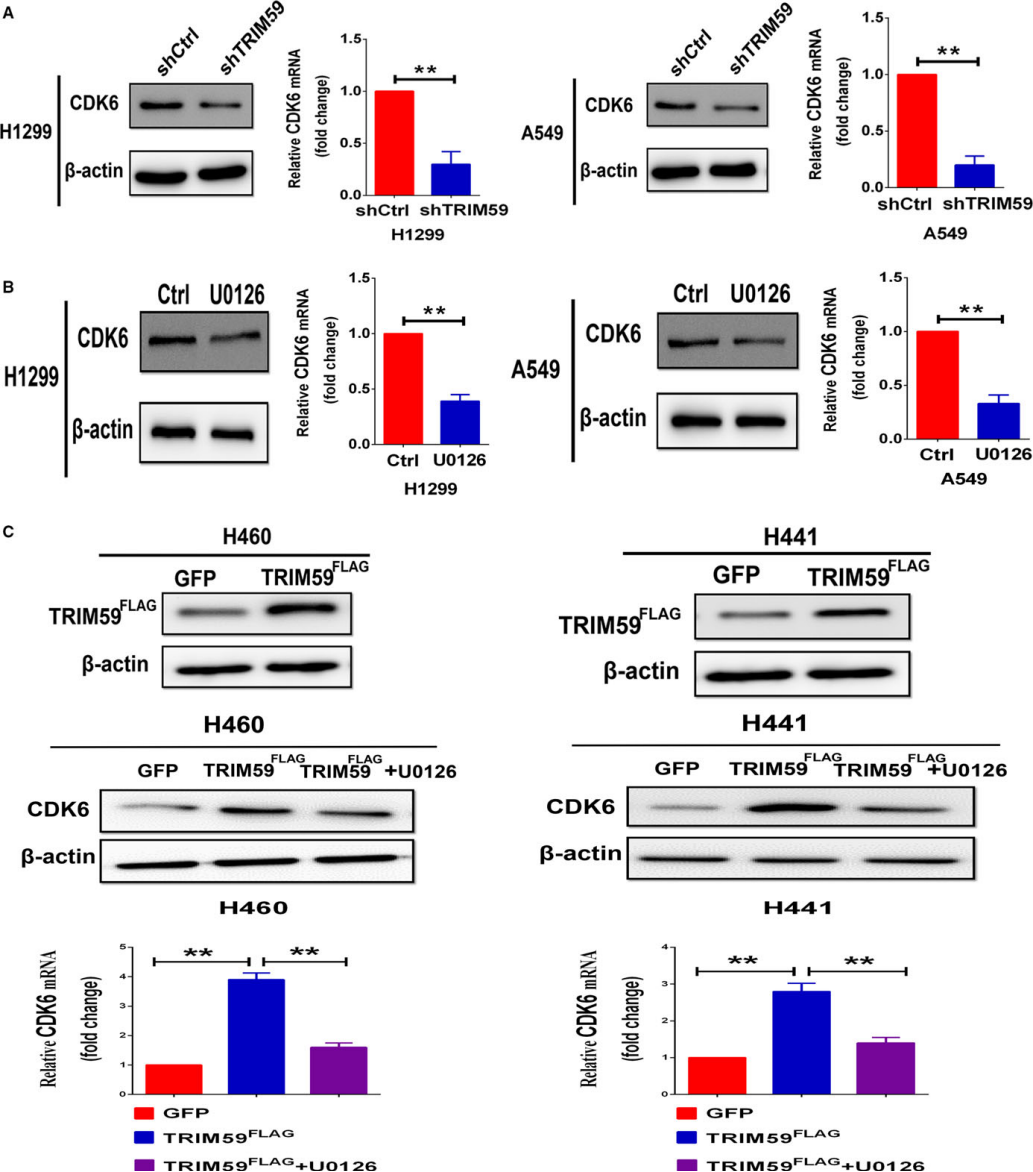
TRIM59 regulates CDK6 expression through ERK signalling pathway. (A) Down‐regulation of TRIM59 significantly suppressed CDK6 mRNA and protein expression in H1299 and A549 cells. (B) Cells were pretreated with negative control or U0126 (10 μmol/L) and were analysed by Western blotting and Quantitative real‐time PCR for CDK6 expression. (C) Confirmation of TRIM59 overexpression in H441 and H460 cells (upper). TRIM59 up‐regulation significantly increased CDK6 gene expression, while the expression of CDK6 was significantly inhibited by ERK inhibitor (lower). Significance was determined by Student's *t* test. *P < 0.05; **P < 0.01

To illustrate the molecular mechanisms of TRIM59 in governing LC cell growth and invasion, in‐depth bioinformatics analysis of the Oncomine database was carried out. The results indicated that the expression of oncoprotein TRIM59 positively correlates with Cyclin CDK6 expression in Wang cell line 2 (A549 cells) and Lee Lung (lung adenocarcinoma and squamous cell lung carcinoma tissue) (Figure 4A,B). As up‐regulation of CDK6 is closely associated with growth and metastasis of different cancers.^11^, ^16^, ^17^, ^18^ This prompted us to test whether TRIM59 promoted cancer growth and metastasis by regulating CDK6 expression through ERK signalling pathway. This speculation is verified by the observations that CDK6 mRNA and protein expression significantly decreased when TRIM59 is down‐regulated by transducing lung cells with TRIM59 shRNA (Figure 5A). Moreover, the expression of CDK6 was also significantly inhibited after treatment of U0126 at 10 μmol/L, which suggested that TRIM59 was involved in the induction of CDK6 expression by phosphorylation of ERK (Figure 5B). To examine the generality of these findings and address whether exogenous up‐regulation of TRIM59 expression via transducing TRIM59^FLAG^ induced the CDK6 expression in TRIM59 low lung cell lines. Remarkably, Exogenous TRIM59 expression in H441 and H460 cells significantly promoted CDK6 gene expression compared with GFP‐treated cells (Figure 5C). As expected, the expression of CDK6 was significantly inhibited by ERK inhibitor in TRIM59 overexpressed cancer cells (Figure 5C). Taken together, these loss‐ and gain‐of‐functional studies demonstrate that TRIM59 can regulate the expression of CDK6 by activation of the ERK pathway.

### 3.5 TRIM59 promoted cancer growth and invasion

**Figure 6:**
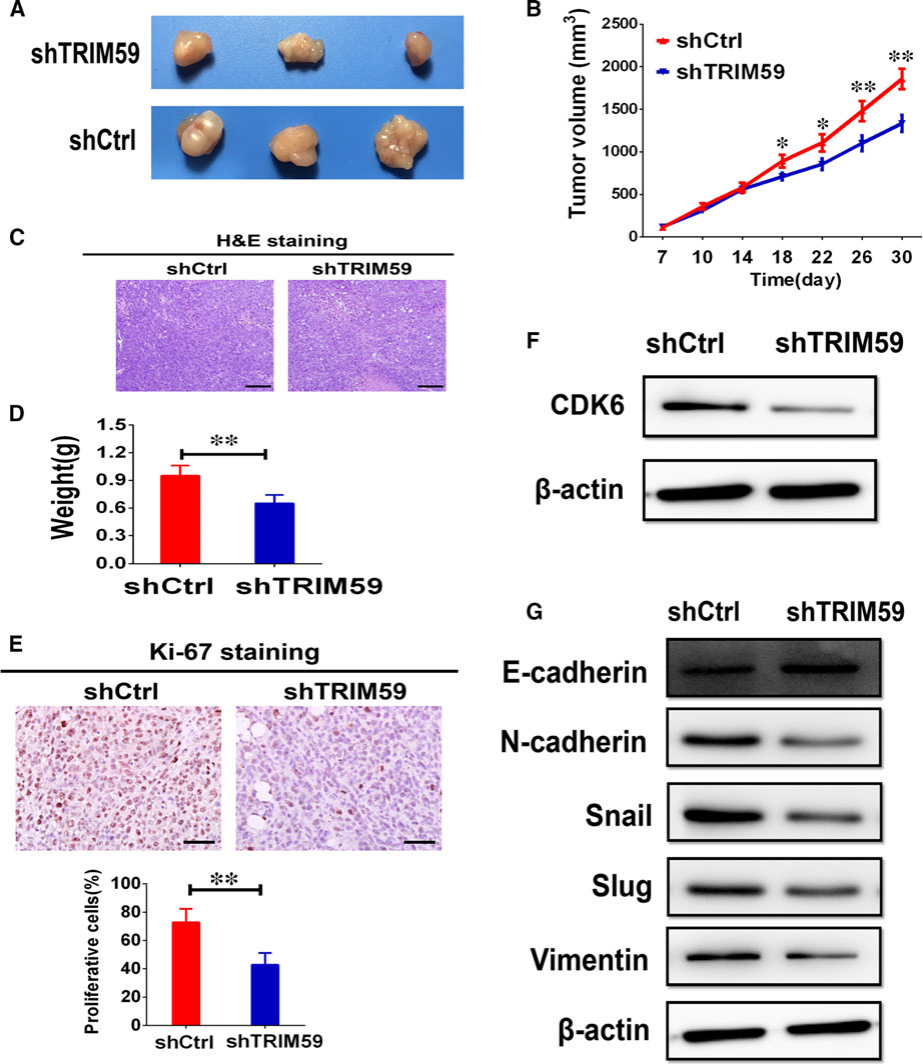
TRIM59 drives tumour growth and metastasis in vivo. (A) Representative images of two xenograft groups. A549 cells tranfected with shCtrl or shTRIM59 plasmid were subcutaneously injected into the back of athymic mice to estimate carcinogenesis. (B) Tumour volumes were measured on the indicated days to assess the effects of TRIM59 on subcutaneous tumour growth. (C) Representative images of tumour samples stained with haematoxylin & eosin (H&E) (200× magnification). (D) The weight of the xenotransplanted tumours in two groups were compared at 30 days postimplantation (n = 8). (E) Representative photos of Ki‐67 immunostaining and statistical analyses of the Ki‐67‐positive index in subcutaneous tumour sections. (F) Proteins of subcutaneous tumours were used for western blotting of CDK6 expression. (G) Western blot evaluations were used to evaluate E‐cadherin, N‐cadherin, Snail, Slug, and Vimentin expression in xenograft tumours. Results are expressed as means ± SEM. The significance was determined by Student's *t* test. *P < 0.05; **P < 0.01

To determine whether TRIM59 governs tumour growth and invasion in vivo, we further examined the effects of TRIM59 expression on in vivo tumour growth of LC cells. We first set up subcutaneous xenograft tumour models by subcutaneously injecting shTRIM59transfected and control shRNA transfected A549 cells in nude mice. Subcutaneous tumour growth was then monitored and compared between the two groups. As shown in Figure 6A,B, the growth of tumour volume by shTRIM59‐transfected cells was significantly suppressed compared with control cells. Likewise, the average weight in the A549 cells with TRIM59 knockdown group were significantly lower than in the control group (Figure 6D). In concordance with the in vitro findings, significantly fewer proliferating cells were observed in the xenografts from shTRIM59‐transfected cells, as indicated by Ki‐67 assay (Figure 6E). By contrast, overexpression of TRIM59 increased the volume and weight of the xenograft tumours and promoted tumour growth in vivo (Figure S1A,C). Moreover, down‐regulation of TRIM59 significantly suppressed the CDK6 expression and EMT program (Figure 6F,G). However, exogenous up‐regulation of TRIM59 expression promoted the CDK6 expression and EMT program (Figure S1D,E). Collectively, these data show that TRIM59 is the critical oncoprotein that mediates LC growth and metastasis.

## 4 DISCUSSION

Metastasis, the major cause of death in LC, is a multistep process involving alterations in the dissemination, invasion, survival, and growth of new cancer cell colonies, which are regulated by a complex network of intra‐ and intercellular signal transduction cascades.^12^ LC metastasis is the main cause of LC‐related mortality. Predicting the clinical outcomes of LC patients is dramatically indispensable and provides a sufficient foundation for personalized cancer treatment. Significant improvements have been made in the prediction of LC outcomes using clinical information, such as TNM staging and several molecular genetic alterations.^2^ However, these factors may not accurately estimate prognosis in LC patients. The heterogeneity of outcomes observed among patients with similar phenotypic properties indicates that genetic variations may play crucial roles in LC growth and metastasis. The present study provides experimental evidence that TRIM59 is overexpressed significantly in LC and the immunostaining intensity of TRIM59 positively correlates with a more aggressive tumour phenotype. Significantly shortened overall survival is seen in patients with high TRIM59 expression compared with those with low TRIM59 expression by Kaplan‐Meier survival curve analysis. Our results suggest that TRIM59 may serve as a valuable new prognostic marker for patients with LC.

To test whether loss of TRIM59 is sufficient to permit cell proliferation and invasion, we then employed TRIM59‐specific shRNA to silence the endogenous TRIM59 expression in A549 and H1299 cells. TRIM59‐knockdown induced by shTRIM59 led to a strong inhibitory effect on proliferation of A549 and H1299 cells. Consistently, we found that stable depletion of TRIM59 in these cells strongly inhibited invasion. Tumour metastasis has been linked to the activation of an EMT‐like program, which involves dissociation of cell‐cell contacts, enhanced migration and dissemination to distant sites.^19^ Recent evidence suggests that EMT acts as an oncogenic modulator of cancer progression in the invasion of tumours including LC. EMT program can be a potential target for therapeutically challenging malignancy.^19^, ^20^ Therefore, understanding the molecular mechanisms of EMT is critical for the treatment of LC. The results of both in vitro and in vivo experiments demonstrate that stable knockdown of TRIM59 inhibited EMT process by increasing E‐cadherin expression and decreasing N‐cadherin, Snail, Slug, and Vimentin expression. Taken together, our results indicate that TRIM59 may mediate the EMT program of LC.

Mitogen‐activated protein kinase is a critical sensor of cellular proliferation and differentiation.^21^ MAPK overexpression or up‐regulation of its activity has been linked to cancer.^21^ MAPK has emerged as a central feature of EMT program in LC.^14^ Moreover, accumulating evidence indicates that ERK activation play an active role in cancer cell growth and invasion through EMT.^22^, ^23^ In this study, we observed that down‐regulation of TRIM59 significantly attenuates ERK signalling in H1299 and A549 cells. In addition, the inhibition of ERK pathway activation significantly suppressed the growth and invasion in these cells. Together, these experiments identify TRIM59‐mediated LC cancer cell growth and invasion is dependent on ERK activation.

Previous studies have suggested that the expression of TRIM59 is related to the cell cycle.^8^ However, little is known about the TRIM59‐related molecules that regulate the cell cycle or their possible roles in cancer cell invasion. The ability to sustain unscheduled proliferation is a hallmark of cancer.^24^ The normal process of cell division occurs via the cell cycle, a series of highly regulated steps that are orchestrated at the molecular level by specific cyclins that act in association with CDKs. CDK4/6 play a key role in cell‐cycle progression by phosphorylating and inactivating the retinoblastoma protein, a tumour suppressor that restrains G1‐to S‐phase progression.^25^ Rapidly emerging data with selective inhibitors of CDK4/6 have validated these cell‐cycle kinases as anticancer drug targets, corroborating longstanding preclinical predictions.^26^, ^27^ In this study, we found that stable depletion of TRIM59 with shRNA in A549 and H1299 cells strongly inhibited CDK6 expression. Moreover, the expression of CDK6 was also potently inhibited by the ERK signalling inhibitor, U0126. Furthermore, exogenous up‐regulation of TRIM59 by transducing TRIM59 low cells with TRIM59^FLAG^, caused significant increases in the CDK6 expression compared with control cells. These results indicate that TRIM59 promotes the expression of CDK6 by regulating ERK activation. Thus, designing inhibitors targeting TRIM59 expression or extra‐regulatory pathway provides a promising approach for conquering LC metastasis.

In summary, our data identify TRIM59 as a key promoter of tumour progression, which uncovers that TRIM59 may become a prognostic marker and therapeutic target for lung carcinoma. Moreover, TRIM59 is involved in lung carcinoma growth and invasion through the induction of CDK6 expression and EMT progression by activation of the ERK pathway.

## CONFLICT OF INTEREST

The authors declare no conflict of interest.

## AUTHOR CONTRIBUTION

B.G. and X.C. involved in the conception and design of the experiments; B.G. and M.L. performed the animal research; M.L., L.Q., X.P., M.L., and H.W. performed the in vitro research; B.G., M.L., W.Z., and L.W. analysed the data; B.G. wrote the paper.
