# Supplementary figures and images for: An TRIM59‐CDK6 axis regulates growth and metastasis of lung cancer
Source: J Cell Mol Med. 2018 Dec 4;23(2):1458–69. doi: 10.1111/jcmm.14052 (PMC6349187; doi:10.1111/jcmm.14052)

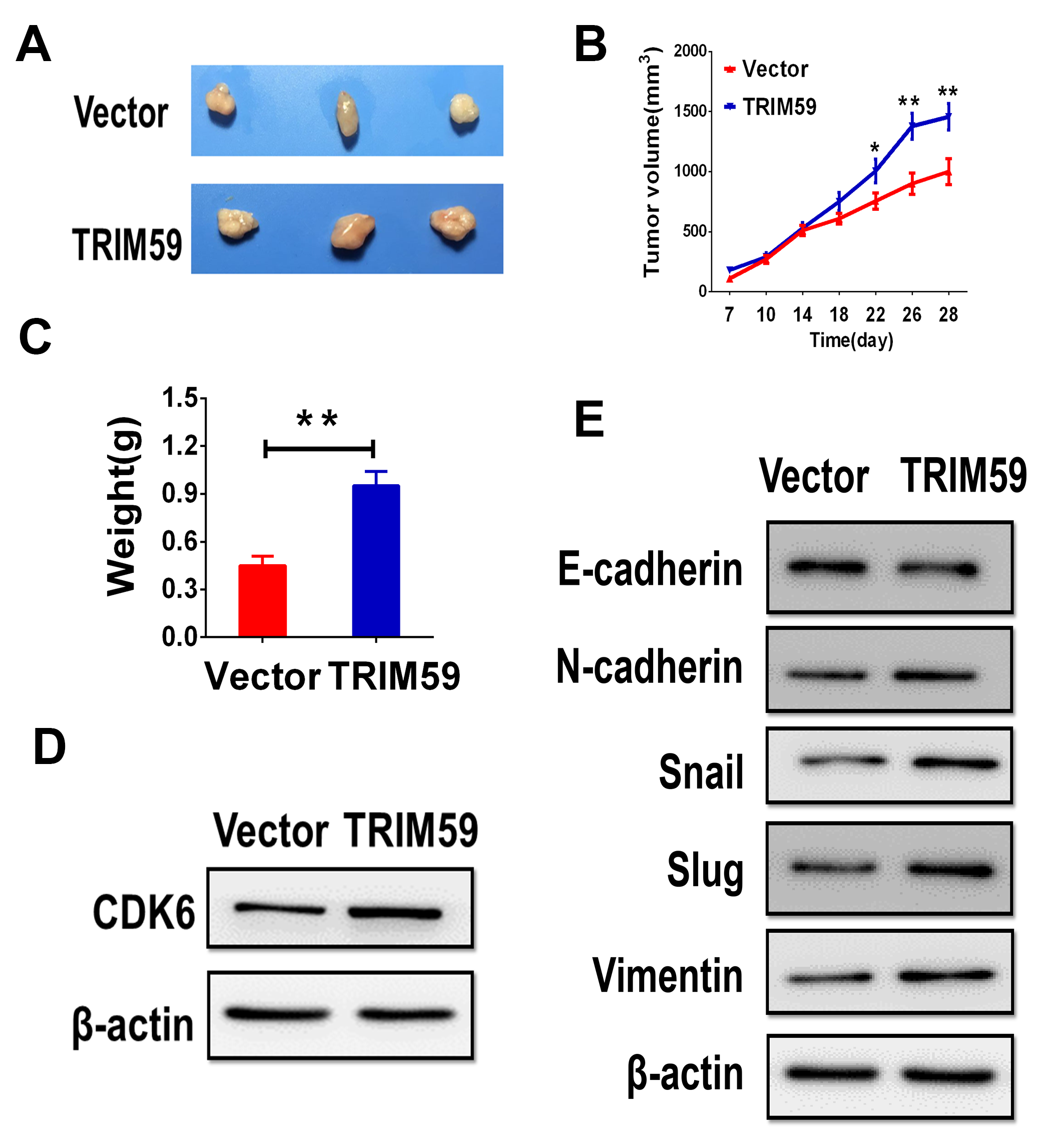

Supplement: Supplementary file 1 [file JCMM-23-1458-s001.tif]
